# Burnout among medical and health sciences information professionals who support systematic reviews: an exploratory study

**DOI:** 10.5195/jmla.2020.665

**Published:** 2020-01-01

**Authors:** Michelle R. Demetres, Drew N. Wright, Antonio P. DeRosa

**Affiliations:** Samuel J. Wood Library, Weill Cornell Medical College, New York, NY, mrd2006@med.cornell.edu; Weill Cornell Medical College, New York, NY, drw2004@med.cornell.edu; Samuel J. Wood Library, Weill Cornell Medical College, New York, NY, apd2004@med.cornell.edu

## Abstract

**Objective:**

The aim of this exploratory study was to assess personal, work-related, and client-related burnout among information professionals who support systematic review (SR) work.

**Methods:**

The Copenhagen Burnout Inventory, a validated tool for assessing burnout, was administered to information professionals who support SR work. A broad range of health sciences or medical librarians and information professionals were targeted via professional email discussion lists and news outlets. Questionnaire responses were captured electronically using Qualtrics Survey Software and quantitatively analyzed.

**Results:**

Respondents experienced an average personal burnout score of 48.6, work-related score of 46.4, and client-related score of 32.5 out of 100. Respondents who reported spending >80% of their job duties on SR work had significantly lower personal burnout scores than those who reported spending <10% of their job duties on SR work (average, 31.5 versus 50.9, respectively). Also, respondents who reported using an SR support tool had significantly lower personal burnout scores than those who reported sometimes using a tool (average, 43.7 versus 54.7, respectively).

**Conclusion:**

The results suggest that information professionals who dedicate more time to SR work or who consistently use an SR support tool experience less burnout. This study provides groundwork for further investigation with the aim of developing approaches to prevent or combat SR-related burnout among information professionals.

## INTRODUCTION

Burnout, defined by Maslach et al. as “a psychological syndrome in response to chronic interpersonal stressors on the job” [1], has long been documented in the health sciences field. This response often manifests itself in three key dimensions: overwhelming exhaustion, feelings of cynicism about or detachment from the job, and a sense of ineffectiveness and/or lack of accomplishment [1].

Burnout and stress have been studied in the field of librarianship in various settings [2–4]. However, considering the growing importance placed on the role of the medical librarian or information professional in creating systematic reviews (SRs), the idea of burnout resulting from SR work has not yet been addressed. Per the Institute of Medicine’s Standards for Systematic Reviews [5], information professionals should be involved in the SR process and are doing so in a variety of different roles, such as consultants and coauthors [6–9]. Nicholson et al. helped identify challenges that information professionals face when working with SR teams [10], recognizing the burden placed on these individuals. Perhaps due in part to the wide range of roles that information professionals play in SRs [11], these projects are often time consuming for information professionals who often have other work outside of SRs [12]. Additionally, much training and many high-level competencies are required for information professionals who are involved in SR work [13, 14]. Taking into consideration these varied roles, challenges, and time and training commitments, this role may contribute to feelings of burnout: “a state of emotional and physical exhaustion that results from long-term involvement in work situations” [15].

However, no studies have yet sought to determine the level of burnout associated with the complexities of contributing to an SR as an information professional. The authors aim to address this gap in the literature by surveying SR information professionals using the validated Copenhagen Burnout Inventory (CBI) to measure burnout [15]. This was a low-risk, voluntary survey. In planning SR services, administrators can potentially use this information to identify avenues to decrease burnout among information professionals. At the least, we hope this study will initiate dialogue concerning the burden involved in SR work.

## METHODS

For this exploratory research study, we administered questionnaires to SR librarians and other information professionals supporting SRs in the medical and health sciences in an effort to examine and measure overall burnout due to SR projects and work. We employed the CBI, a freely available, validated tool for assessing burnout because of its explicit adaptability to different disciplines, which was therefore valid for use among librarians [15].

This tool is compartmentalized into 3 distinct scales: (1) personal burnout, (2) work-related burnout, and (3) client-related burnout. The authors of the CBI used Maslach et al.’s definition of burnout and built upon it with these 3 domains. These 3 scales uniquely reflect different aspects involved in SR work: the personal aspect, the SR work itself, and collaboration with SR client teams. The CBI uses a rating system that measures responses on a scale of 0–100, with 0 indicating no burnout and 100 indicating the highest level of burnout. In addition to the CBI, we included demographic and occupational questions to capture granular information regarding our respondents. The full survey tool is available in supplemental Appendix A.

We targeted libraries and information centers via three professional email discussion lists and news outlets: MEDLIB-L, DOCLINE, and the Medical Library Association’s (MLA’s) *MLA News.* Any individual subscribing to these lists, including nonlibrarians and paraprofessionals, could participate in the survey. This study was classified as exempt by the Weill Cornell Medicine Institutional Review Board (protocol # 1807019460). The survey was left open to responses for 1 month and closed on October 1, 2018. Questionnaire responses were captured electronically using Qualtrics Survey Software [16]. At the close of the survey, data were coded in Excel according to the CBI scale guidelines. Quantitative analysis was undertaken using the scoring metric outlined in the CBI, and results were synthesized using qualitative techniques and statistical methods. Power calculations and statistical analyses were performed using R 3.5.1 [17]. TukeyHSD and Kruskal-Wallis tests in R [17] were used to calculate differences in burnout scores between the following respondent or demographic groups: <10% versus >80% of job duties devoted to SR work, research versus reference librarians, solo versus non-solo librarians, and uses versus sometimes uses an SR support tool.

## RESULTS

The questions, scoring, and response frequencies of the 3 CBI scales are shown in Table 1. Out of a total of 198 respondents who initiated the survey, 166 qualified as completed for the personal burnout scale with 2 questions unanswered by 2 respondents; 159 qualified as completed for the work burnout scale with no questions unanswered; and 151 qualified as completed for the client burnout scale with 3 questions unanswered by 4 respondents. Qualifying responses were determined for the respondent level based on the rules of the CBI:

**Table 1:**
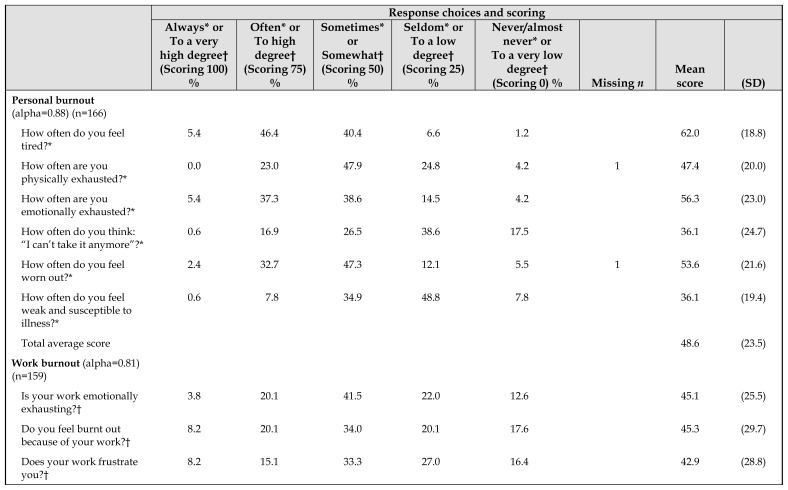

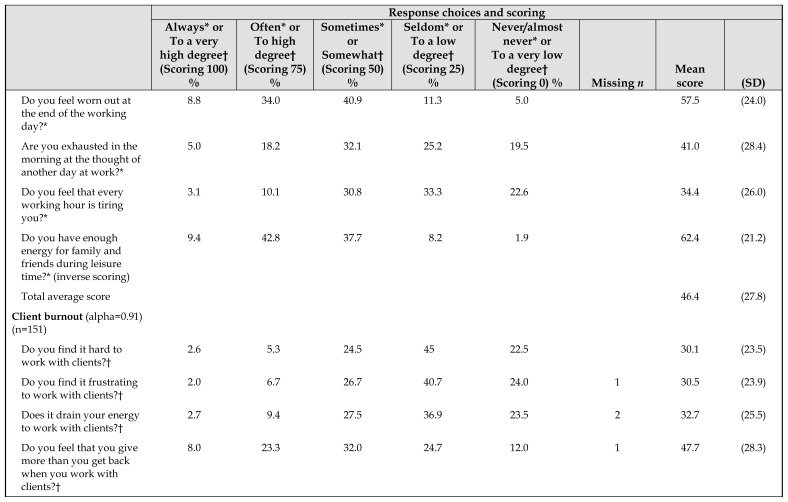
Copenhagen Burnout Inventory (CBI) items and scores

	Response choices and scoring							
	Always* or To a very high degree† (Scoring 100) %	Often* or To high degree† (Scoring 75) %	Sometimes* or Somewhat† (Scoring 50) %	Seldom* or To a low degree† (Scoring 25) %	Never/almost never* or To a very low degree†(Scoring 0) %	Missing *n*	Mean score	(SD)
**Personal burnout** (alpha=0.88) (n=166)								
How often do you feel tired?*	5.4	46.4	40.4	6.6	1.2		62.0	(18.8)
How often are you physically exhausted?*	0.0	23.0	47.9	24.8	4.2	1	47.4	(20.0)
How often are you emotionally exhausted?*	5.4	37.3	38.6	14.5	4.2		56.3	(23.0)
How often do you think: “I can’t take it anymore”?*	0.6	16.9	26.5	38.6	17.5		36.1	(24.7)
How often do you feel worn out?*	2.4	32.7	47.3	12.1	5.5	1	53.6	(21.6)
How often do you feel weak and susceptible to illness?*	0.6	7.8	34.9	48.8	7.8		36.1	(19.4)
Total average score							48.6	(23.5)
**Work burnout** (alpha=0.81) (n=159)								
Is your work emotionally exhausting?†	3.8	20.1	41.5	22.0	12.6		45.1	(25.5)
Do you feel burnt out because of your work?†	8.2	20.1	34.0	20.1	17.6		45.3	(29.7)
Does your work frustrate you?†	8.2	15.1	33.3	27.0	16.4		42.9	(28.8)
Do you feel worn out at the end of the working day?*	8.8	34.0	40.9	11.3	5.0		57.5	(24.0)
Are you exhausted in the morning at the thought of another day at work?*	5.0	18.2	32.1	25.2	19.5		41.0	(28.4)
Do you feel that every working hour is tiring you?*	3.1	10.1	30.8	33.3	22.6		34.4	(26.0)
Do you have enough energy for family and friends during leisure time?* (inverse scoring)	9.4	42.8	37.7	8.2	1.9		62.4	(21.2)
Total average score							46.4	(27.8)
**Client burnout** (alpha=0.91) (n=151)								
Do you find it hard to work with clients?†	2.6	5.3	24.5	45	22.5		30.1	(23.5)
Do you find it frustrating to work with clients?†	2.0	6.7	26.7	40.7	24.0	1	30.5	(23.9)
Does it drain your energy to work with clients?†	2.7	9.4	27.5	36.9	23.5	2	32.7	(25.5)
Do you feel that you give more than you get back when you work with clients?†	8.0	23.3	32.0	24.7	12.0	1	47.7	(28.3)
Are you tired of working with clients?*	2.0	4.6	31.1	31.8	30.5		29.0	(24.5)
Do you sometimes wonder how long you will be able to continue working with clients?*	0.7	9.9	24.5	22.5	42.4		26.0	(26.6)
Total average score							32.5	(26.3)

Score range for all categories/questions is 0–100.

* Response choices for questions denoted with ^*^.

† Response choices for questions denoted with ^†^.

Personal burnout=if fewer than three questions have been answered, the respondent is classified as non-responder; work burnout=if fewer than four questions have been answered, the respondent is classified as non-responder; client burnout=if fewer than three questions have been answered, the respondent is classified as non-responder. [15]

Overall, respondents had an average personal burnout score of 48.6, a work-related burnout score of 46.4, and a client-related burnout score of 32.5. When asked if they were emotionally exhausted in terms of personal burnout, most respondents (81.3%) answered at least “sometimes.” Most respondents (83.7%) also answered at least “sometimes” when asked if they felt worn out at the end of the working day. When respondents were asked if they felt that they give more than they get back when working with clients, 63.3% of respondents answered at least “somewhat.”

Based on reported job titles, responses were grouped into 4 broader categories: research librarian, reference librarian, clinical librarian, and an “other” group. The other group included individuals with the following job titles: director, manager, public/outreach services, information specialist, document delivery service/interlibrary loan, technical services, informationist, methodologist, guidelines developer, and SR coordinator. Regrouping was done based on agreement of 2 independent reviewers. Table 2 contains the average burnout scores by job title group. Reference librarians (n=27), with an average total burnout score of 47.1, reported higher levels of burnout across all 3 CBI scales than all other types of information professionals. Research librarians (n=32) consistently reported the lowest levels of burnout across the 3 scales, with an average total burnout score of 37.7.

**Table 2:** Average CBI burnout scores by job title

Job	Personal burnout		Work burnout		Client burnout	

	no. of respondents	Score	no. of respondents	Score	no. of respondents	Score
Reference librarian	27	52.0	27	52.0	27	37.2
Other	50	50.2	50	48.9	50	34.3
Clinical librarian	33	46.0	33	43.9	33	32.3
Research librarian	32	45.7	32	40.6	32	26.7
Average		48.5		46.4		32.6

Regarding the number of years supporting SRs, respondents with 7–10 years of experience (n=18) had the highest average total burnout score (i.e., average of personal, work, and client scores) of 51.4. Respondents with fewer (<1 year, n=17; 1–2 years, n=29; 3–6 years, n=56) or more (10+ years, n=23) years of experience had lower scores, ranging from 41.1 to 42.1. Notably, individuals with 10+ years of experience had the widest spread of scores, with the lowest client-related burnout score (27.7) and the second highest personal burnout score (51.4).

Regarding the number of hours spent working on SRs, respondents who spent the most time on SRs (20+ hours, n=36) had the lowest average total burnout score of 39.6. Those spending 11–15 hours (n=34) had the highest score at 45.0. Those spending 16–20 hours (n=22), 1–5 hours (n=30), and 6–10 hours (n=19) had scores of 43.6, 43.5, and 39.7, respectively.

Regarding use of an SR support tool (e.g., Covidence, Distiller SR, Rayyan), respondents who reported using a tool (n=49) had the lowest average total burnout score of 38.5. Those who reported sometimes using a tool (n=25) had the highest score of 46.4, and those reporting no use of a tool (n=68) had a lower score of 43.2.

**Figure 1:**
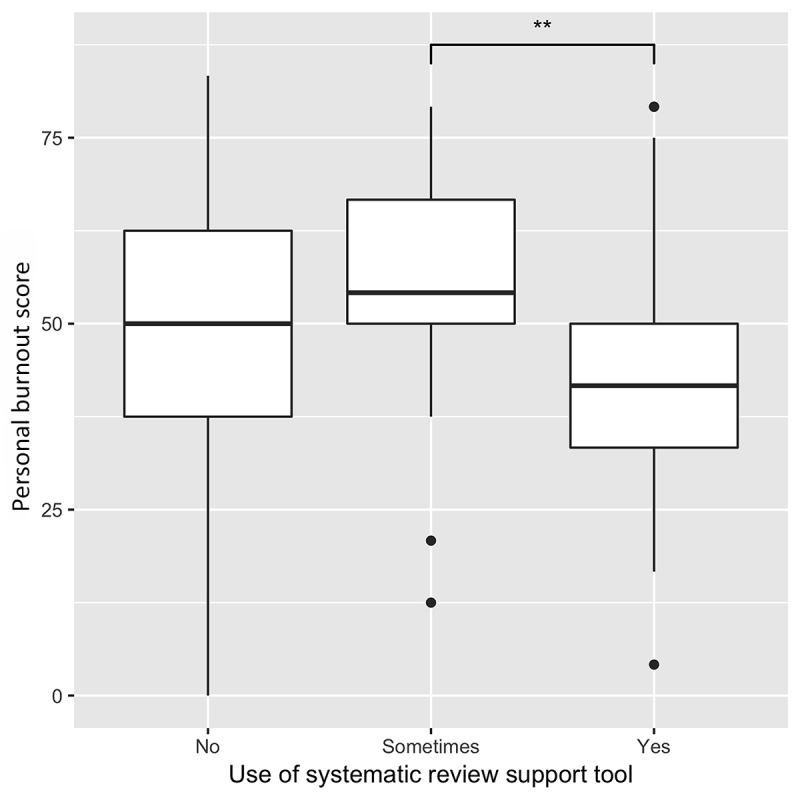
Personal burnout score depending on use of a systematic review (SR) support tool

**Figure 2:**
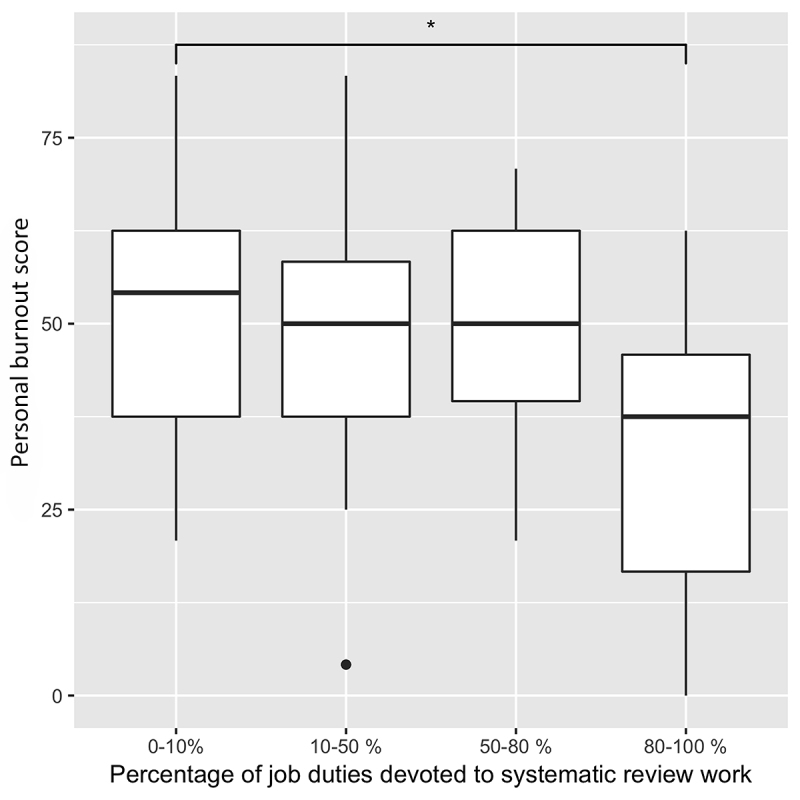
Personal burnout score depending on the percentage of job duties devoted to SR work

Statistical analysis showed that respondents who reported using a SR support tool had significantly lower personal burnout scores than those who reported sometimes using a tool (*p*=0.021) (Figure 1). Also, respondents who reported spending >80% of their job duties on SRs had significantly lower personal burnout scores than those who reported spending <10% of their job duties on SRs (*p*=0.007) (Figure 2). All other comparisons were nonsignificant (*p*>0.05).

## DISCUSSION

With overall average personal and work-related burnout scores nearing fifty, our findings are consistent with previous studies reporting high levels of burnout among workers in service-oriented occupations, which can lead to potential emotional exhaustion, depersonalization, and diminished personal accomplishments at work [18–20]. When analyzing the survey results across all three CBI scales, we attempted to categorize and rank respondent subgroups by their job titles. However, due to an insufficient number of respondents, no statistically significant differences among job titles could be detected. However, we did note some trends, such as reference librarians experiencing more burnout than clinical or research librarians. Several factors potentially contribute to burnout for certain types of information professionals. For instance, how thinly these individuals were spread in terms of the number of SRs they worked on simultaneously might be a good indicator of the potential to experience burnout and to improve experiences with SR clients. Spencer and Eldredge’s identification of the varying roles that different types of information professionals play in SRs beyond simply searching the literature could be a starting point for further exploration into these three job subgroups and could be beneficial to better understanding their needs [11].

In regard to demographic and occupational responses, for the most part, disparate sample sizes for the individual groups and statistically nonsignificant differences make drawing conclusions untenable. However, we did find a significant difference in personal burnout score based on dedicated SR work time. Information professionals with >80% of their job duties devoted to SR work reported lower personal burnout than those who spent less time on SR work. This suggests that dedicated time solely for SR work can reduce burnout. However, when individuals have more varied job duties outside of SR work and, thus, less time to devote to each step in the SR process, this might contribute to more burnout. We also found that information professionals who consistently used an SR support tool reported lower personal burnout than those who only sometimes used such a tool. Consistently using a tool might help streamline the SR process and overcome the initial anxieties involved in learning to use new software, as shown in previous literature [21, 22]. Conversely, inconsistently using a tool could complicate an already difficult workflow when juggling several projects and SR support processes (e.g., End Note, Excel).

A major limitation of this study was that a robust analysis of job titles was not possible due to the large number and unequal distribution of job titles reported, many of which had potentially overlapping work responsibilities. Future studies should more clearly define different types of job titles and target underrepresented groups to obtain less skewed results and more statistical power. Also, as with many emailed surveys, voluntary response bias was possible. In particular for this survey, individuals experiencing burnout might have been more likely to respond. Future studies may need to administer surveys to a representative sample to mitigate bias. Another limitation of this study is that we could not be certain that respondents answered the survey considering only their SR work. We recognize that it is difficult to compartmentalize one’s job functions with regard to burnout; however, we attempted to make this as explicit as possible in the call for participation in the study. We also noted that racial and ethnic diversity among respondents was almost nonexistent. With 92% of respondents self-identifying as white, other racial groups were underrepresented. This is not unique to this study but reflects in the profession at large [23]. It also may be interesting to investigate whether librarian coauthorship on published SRs influences their burnout scores.

Our results suggest that having a dedicated SR librarian or information professional could be beneficial for combating SR-related burnout in the profession. In addition, consistently using an SR support tool may help mitigate personal burnout.

## SUPPLEMENTAL FILES

Appendix A Systematic review burnout inventory

Appendix B R script to determine Cronbach alphas

## 

**Michelle R. Demetres**, mrd2006@med.cornell.edu, https://orcid.org/0000-0002-4997-7707, Samuel J. Wood Library, Weill Cornell Medical College, New York, NY

**Drew N. Wright**, drw2004@med.cornell.edu, https://orcid.org/0000-0002-1776-5427, Weill Cornell Medical College, New York, NY

**Antonio P. DeRosa**, AHIP, apd2004@med.cornell.edu, https://orcid.org/0000-0003-3518-0497, Samuel J. Wood Library, Weill Cornell Medical College, New York, NY
