# Supplementary material for: Burnout among medical and health sciences information professionals who support systematic reviews: an exploratory study
Source: J Med Libr Assoc. 2020 Jan 1;108(1):89–97. doi: 10.5195/jmla.2020.665 (PMC6919998; doi:10.5195/jmla.2020.665)
Supplement: Appendix A [file jmla-108-89-s001.pdf]

JMLA Journal of the Medical Library Association

Supplemental content to
J Med Libr Assoc. Jan;108(1):dx.doi.org/10.5195/jmla.2020.665
www.jmla.mlanet.org
© Demetres, Wright, DeRosa 2020

Burnout among medical and health sciences information professionals who support systematic reviews: an exploratory study

Michelle R. Demetres; Drew N. Wright; Antonio P. DeRosa, AHIP

APPENDIX A

Systematic review burnout inventory

The Samuel J. Wood Library at Weill Cornell Medicine is conducting a study about burnout among medical and health sciences information professionals who support systematic review research. We invite you to participate in a brief survey about your level of personal, professional, and client-related burnout in supporting systematic review research. We are requesting participation from all information workers, regardless of your level or type of involvement in supporting systematic reviews. This survey should take less than ten minutes of your time. The questions will be at a general level and will not ask for protected or any identifying information. By participating, you will help us assess the level of burnout and identify themes among information professionals' systematic review work. Participating in this study is voluntary, and participants will remain completely anonymous. Even if you decide to participate now, you may change your mind and stop at any time. You may choose not to answer any survey question for any reason. This survey and research study have been declared exempt by the Weill Cornell Medicine Institutional Review Board (protocol #1807019460).

The survey will be open until Monday, October 1, 2018.

Please click the arrow button below to begin.

Part 1: Personal burnout

Q1 How often do you feel tired?
o Always
o Often
o Sometimes
o Seldom
o Never/almost never

Q2 How often are you physically exhausted?
o Always
o Often
o Sometimes
o Seldom
o Never/almost never

Q3 How often are you emotionally exhausted?
o Always
o Often
o Sometimes
o Seldom
o Never/almost never

Journal of the Medical Library Association
Supplemental content to
J Med Libr Assoc. Jan;108(1):dx.doi.org/10.5195/jmla.2020.665
www.jmla.mlanet.org
© Demetres, Wright, DeRosa 2020

Q4 How often do you think: "I can't take it anymore"?
- Always
- Often
- Sometimes
- Seldom
- Never/almost never

Q5 How often do you feel worn out?
- Always
- Often
- Sometimes
- Seldom
- Never/almost never

Q6 How often do you feel weak and susceptible to illness?
- Always
- Often
- Sometimes
- Seldom
- Never/almost never

Part 2: Work-related burnout

Where applicable, please answer the following as they relate to your systematic review work only.

Q7 Is your work emotionally exhausting?
- To a very high degree
- To a high degree
- Somewhat
- To a low degree
- To a very low degree

Q8 Do you feel burnt out because of your work?
- To a very high degree
- To a high degree
- Somewhat
- To a low degree
- To a very low degree

Q9 Does your work frustrate you?
- To a very high degree
- To a high degree
- Somewhat
- To a low degree
- To a very low degree

Q10 Do you feel worn out at the end of the working day?
- Always
- Often
- Sometimes
- Seldom
- Never/almost never

JMLA Journal of the Medical Library Association

Supplemental content to
J Med Libr Assoc. Jan;108(1):dx.doi.org/10.5195/jmla.2020.665
www.jmla.mlanet.org
© Demetres, Wright, DeRosa 2020

Q11 Are you exhausted in the morning at the thought of another day at work?
- o Always
- o Often
- o Sometimes
- o Seldom
- o Never/almost never

Q12 Do you feel that every working hour is tiring you?
- o Always
- o Often
- o Sometimes
- o Seldom
- o Never/almost never

Q13 Do you have enough energy for family and friends during leisure time?
- o Always
- o Often
- o Sometimes
- o Seldom
- o Never/almost never

Part 3: Client-related burnout

Where applicable, please answer the following as they relate to your systematic review work only.

Q14 Do you find it hard to work with clients?
- o To a very high degree
- o To a high degree
- o Somewhat
- o To a low degree
- o To a very low degree

Q15 Do you find it frustrating to work with clients?
- o To a very high degree
- o To a high degree
- o Somewhat
- o To a low degree
- o To a very low degree

Q16 Does it drain your energy to work with clients?
- o To a very high degree
- o To a high degree
- o Somewhat
- o To a low degree
- o To a very low degree

Q17 Do you feel that you give more than you get back when you work with clients?
- o To a very high degree
- o To a high degree
- o Somewhat
- o To a low degree
- o To a very low degree

JMLA Journal of the Medical Library Association

SC
Supplemental content to
J Med Libr Assoc. Jan;108(1):dx.doi.org/10.5195/jmla.2020.665
www.jmla.mlanet.org
© Demetres, Wright, DeRosa 2020

Q18 Are you tired of working with clients?
- Always
- Often
- Sometimes
- Seldom
- Never/almost never

Q19 Do you sometimes wonder how long you will be able to continue working with clients?
- Always
- Often
- Sometimes
- Seldom
- Never/almost never

Part 4: Demographics

Q20 What is your job title?
- Reference librarian
- Scholarly communications librarian
- Clinical librarian
- Technical services librarian
- Electronic resources librarian
- Instructional librarian
- Research librarian
- Director
- Assistant director
- Associate director
- Manager
- Student
- Other ____

Q21 Are you a solo librarian?
- Yes
- No

Q22 What is your age?
- 18–24 years old
- 25–34 years old
- 35–44 years old
- 45–54 years old
- 55–64 years old
- 65–74 years old
- 75 years or older

Q23 Please specify your ethnicity origin or race
- White
- Black or African American
- Hispanic or Latinx
- Asian or Pacific Islander
- Native American
- Other ____

JMLA Journal of the Medical Library Association

Supplemental content to
J Med Libr Assoc. Jan;108(1):dx.doi.org/10.5195/jmla.2020.665
www.jmla.mlanet.org
© Demetres, Wright, DeRosa 2020

Q24 For how many years have you been supporting systematic reviews?
- o < 1 year
- o 1–2 years
- o 3–6 years
- o 7–10 years
- o 10+ years

Q25 What percentage of your job duties is devoted to systematic review work?
- o <10%
- o 10%–50%
- o 50%–80%
- o >80%

Q26 In which stages of the systematic review process are you typically involved? (Please select all that apply)
- o Consultation
- o Education/instruction
- o Term harvesting
- o Search strategy development
- o Database searching
- o Citation management
- o Reviewing/abstracting phases
- o Critical appraisal
- o Full text retrieval
- o Manuscript writing
- o Other ______________________________

Q27 What types of users do you service in regard to systematic reviews? (Please select all that apply)
- o Students
- o Faculty
- o Physicians
- o Residents
- o Fellows
- o Nurses
- o Allied health professionals
- o Administration/leadership
- o Other ______________________________

Q28 How many hours (on average) do you typically spend actively working on or supporting a single systematic review project? (Includes any/all aspects of the systematic review process.)
- o 1–5 hours
- o 6–10 hours
- o 11–15 hours
- o 16–20 hours
- o 20+ hours

Q29 Do you use a systematic review support tool (Covidence, Distiller SR, Rayyan, etc.)?
- o Yes
- o No
- o Sometimes

JMLA Journal of the Medical Library Association

SC
Supplemental content to
J Med Libr Assoc. Jan;108(1):dx.doi.org/10.5195/jmla.2020.665
www.jmla.mlanet.org
© Demetres, Wright, DeRosa 2020

Q30 How many information professionals typically work together on a single systematic review project at your institution?
- o No partnership (zero)
- o 2
- o More than 2
