# Supplementary material for: Burnout among medical and health sciences information professionals who support systematic reviews: an exploratory study
Source: J Med Libr Assoc. 2020 Jan 1;108(1):89–97. doi: 10.5195/jmla.2020.665 (PMC6919998; doi:10.5195/jmla.2020.665)
Supplement: Appendix B [file jmla-108-89-s002.pdf]

JMLA Journal of the Medical Library Association

SC

Supplemental content to
J Med Libr Assoc. Jan;108(1):dx.doi.org/10.5195/jmla.2020.665
www.jmla.mlanet.org
© Demetres, Wright, DeRosa 2020

Burnout among medical and health sciences information professionals who support systematic reviews: an exploratory study

Michelle R. Demetres; Drew N. Wright; Antonio P. DeRosa, AHIP

APPENDIX B

R script to determine Cronbach alphas

```
library(psych)

burnout_alpha_personal <- read_csv("/burnout_alpha_personal.csv")
burnout_alpha_work <- read_csv("/burnout_alpha_work.csv")
burnout_alpha_client <- read_csv("/burnout_alpha_client.csv")

bapdat <-data.matrix(burnout_alpha_personal)
bawdat <-data.matrix(burnout_alpha_work)
bacdat <-data.matrix(burnout_alpha_client)

alpha(bapdat)
alpha(bawdat)
alpha(bacdat)

*R script to determine AOV and Boxplot*

library(rlang)
library(ggplot2)
library(ggsignif)

burnout_percentage <- read_csv("/burnout-percentage.csv")

TukeyHSD(aov(formula = Personal ~ Response, data = burnout_percentage))
TukeyHSD(aov(formula = Work ~ Response, data = burnout_percentage))
TukeyHSD(aov(formula = Client ~ Response, data = burnout_percentage))

kruskal.test(Work ~ as.factor(Response), data = burnout_percentage)
kruskal.test(Personal ~ as.factor(Response), data = burnout_percentage)
kruskal.test(Client ~ as.factor(Response), data = burnout_percentage)

ggplot(burnout_percentage, aes(Response, Personal)) + geom_boxplot() + geom_signif(comparisons =
list(c("1", "4")), map_signif_level=TRUE) + labs(y = "Personal Burnout Score", x = "Response Group")
```
